# Synthesis and Evaluation of Antioxidant and Potential Prebiotic Activities of Acetylated and Butyrylated Fructo-Oligosaccharides

**DOI:** 10.3390/antiox11091658

**Published:** 2022-08-26

**Authors:** Shuxin Zhou, Wei Zhu, Xianjin Qin, Shipo Li, Weihua Chu

**Affiliations:** 1 State Key Laboratory of Natural Medicines, School of Life Science and Technology, China Pharmaceutical University, Nanjing 210009, China; 2 School of Pharmaceutical Science, Peking University, Beijing 100089, China

**Keywords:** FOS esterification, SCFAs, antioxidant activity, prebiotic properties

## Abstract

Fructo-oligosaccharides (FOS) have well-known bifidogenic effects as probiotics. In this study, esterification was adopted for FOS modification to produce better prebiotic properties. We synthesized and characterized acetylated fructo-oligosaccharides (Ac-FOS) and butyrylated fructo-oligosaccharides (Bu-FOS) as candidate prebiotics. Antioxidant activity and prebiotic esactiviti were evaluated as important indicators. We found, surprisingly, that butyrylation was an effective method in significantly improving the antioxidant activity of FOS. The fermentation products of feces from mice added to Ac-FOS and Bu-FOS, were investigated in vitro, including changes of pH values, short-chain fatty acids (SCFAs) production, and microbiota composition. Supplementation of Ac-FOS or Bu-FOS increased pH values and promoted the growth and activity of beneficial intestinal bacteria, such as *Bifidobacteria* and *Lactobacillus*. More importantly, the levels of prebiotic SCFAs were obviously elevated as detected by Gas Chromatography–Mass Spectrometry (GC-MS). Results suggest that Ac-FOS and Bu-FOS have great potential applications in SCFA delivery systems and gut microbiota regulation.

## 1. Introduction

Ingestion of plant-derived prebiotics to alter the gut microbiota and improve human health is a spectrum of therapeutic strategies [1]. Fructo-oligosaccharides are well known as water-soluble dietary fibers, and have been attracting interest as prebiotic ingredients [2]. FOS resist intestinal digestion by digestive enzymes in the upper GIT and reach the large intestine without structural change [3,4]. Numerous studies have indicated that FOS is selectively consumed by probiotic bacteria such as bifidobacteria and lactobacilli species, favorably promotes their growth, improves the production of short-chain fatty acids, and further advances well-balanced gut microbiota [5,6,7,8]. Intake of FOS also has physiological effects on distant site organs such as the liver, heart, brain, pancreas, and others [5]. Owing to the special and important biological activities of prebiotics, including antioxidant, antibacterial, and immunological activities, chemical modifications are used frequently as common methods to enhance biological functions and find new agents with medicinal values. Different modification methods of plant-based prebiotics include sulfation, phosphorylation, carboxymethylation, selenization, methylation, acetylation, ammunition, amidation, iodization, sulfonylation, hydroxypropylation, and carbonylation, which alter one or more traits. For example, phosphorylated polysaccharides have demonstrated high scavenging ability on hydroxyl radicals and growth-promoting activity of *Lactobacillus bulgaricus*. Carboxymethylated polysaccharides show the stronger DPPH and ABTS radical scavenging effects.

Acetylation and butyrylation of polysaccharides have been reported in previous studies. Acetylated polysaccharides display the best proliferation effects on *Bifidobacterium adolescentis* [9]. Butyl group distribution plays a pivotal role in regulating the intestinal digestion and colonic fermentation of butyrylated starch [10]. Acetylated laminarin was synthesized and exhibited antiviral activity [11]. Wampee fruit peel pectin by acetic had better functional properties, such as antioxidant activity and promoting probiotics ability [12]. Acetylated polysaccharides from *Pleurotus geesteranus* showed anti-inflammatory, antioxidant, and lung protection effects [13]. Butyrylated inulin, kraft lignin and lignosulfonate have also been reported [14,15,16].

Thus, we speculated that the chemical modification of FOS could change bioactivities and even result in more effective prebiotics. Acetylated and butyrylated fructo-oligosaccharides were synthesized and characterized for validation of the idea. More importantly, biological evaluations of two synthetic candidate prebiotics were performed to explain their regulation mechanisms and application directions.

## 2. Materials and Methods

### 2.1. Materials

Commercial FOS were purchased from Qingdao Century Longlive International Trade Co., Ltd. (Qingdao, China) and were of >90% purity. All other chemicals were purchased from Sigma-Aldrich Co., Inc. (St. Louis, MO, USA). Bacterial culture media and other additives were obtained from Oxoid (Basingstoke Hampshire, UK).

### 2.2. Bacterial Strain

*Lactobacillus rhamnosus* (CGMCC1.3724) and *Bifidobacterium longum* (CGMCC1.2186) were used in the present study, and were isolated from traditional Chinese fermented foods and identified by API 50 CHL kit (bioMérieux Inc., Marcy l’Etoile, France) and 16S rDNA sequencing analysis. The stock culture was maintained in Man Rogosa Sharpe Medium (MRS medium) with 20% glycerol and stored at −80 °C.

### 2.3. Synthesis and Characterization of Esterified Fructo-Oligosaccharides

#### 2.3.1. Synthesis of Ac-FOS and NMR Analysis

Available commercial FOS (5 g) was dissolved in pyridine (30 mL), and then the solution was cooled to 0 °C, followed by the addition of acetic anhydride (5 mL) and 4-dimethylaminopyridine (DMAP, 100 mg). The reaction solution was stirred under an argon atmosphere at 25 °C for 8 h. Saturated aqueous NaHCO_3_ (20 mL) was added to the solution to quench the reaction after TLC indicated the completion of the reaction [17]. The solvent was removed in vacuo, then the residue was dissolved in dichloromethane (100 mL). The solution was washed with 1N HCl and saturated NaCl aqueous three times, respectively, and then dried with Na_2_SO_4_. The solvent was removed in vacuo and the residue was purified by flash chromatography on silica gel (at the gradient of 52% ethyl acetate in petroleum ether and ethyl acetate elution system) to give Ac-FOS 5.4 g as a colorless oil.

The synthesized Ac-FOS was analyzed by 400 MHz ^1^H nuclear magnetic resonance (NMR) and 101 MHz ^13^C NMR.

^1^H NMR (400 MHz, CDCl_3_) δ 5.75 (d, *J* = 3.8 Hz, 1H), 5.69 (d, *J* = 7.9 Hz, 1H), 5.45 (ddd, *J* = 20.0, 11.6, 8.4 Hz, 3H), 5.35 (t, *J* = 6.5 Hz, 1H), 5.07 (t, *J* = 9.8 Hz, 1H), 4.91 (dd, *J* = 10.4, 3.8 Hz, 1H), 4.42–4.09 (m, 11H), 3.67 (dd, *J* = 24.9, 9.6 Hz, 2H), 2.16–2.00 (m, 33H, COCH_3_).

^13^C NMR (101 MHz, CDCl_3_) δ 170.62 (C=O), 170.55 (C=O), 170.42 (C=O), 169.99(C=O*4), 169.89 (C=O), 169.67(C=O *2), 169.50 (C=O), 103.43, 102.94, 89.29, 78.40, 77.80, 77.27, 76.58, 75.51, 74.99, 73.75, 70.03, 69.82, 68.26, 68.24, 63.69, 63.19, 62.68, 62.25, 61.71, 20.71 (CH_3_), 20.67 (CH_3_), 20.61 (CH_3_), 20.56 (CH_3_), 20.53 (CH_3_), 20.44 (CH_3_).

#### 2.3.2. Synthesis of Bu-FOS and NMR Analysis

FOS (5 g) was dissolved in pyridine (30 mL), and then the solution was cooled to 0 °C, followed by the addition of butyric anhydride (5 mL) and 4-dimethylaminopyridine (DMAP, 100 mg). The reaction solution was stirred under an argon atmosphere at 25 °C for 14 h. Saturated aqueous NaHCO_3_ (20 mL) was added to the solution to quench the reaction after TLC indicated the completion of the reaction [18]. The solvent was removed in vacuo, then the residue was dissolved in dichloromethane (100 mL). The solution was washed with 1 N HCl and saturated NaCl aqueous three times, respectively, and then dried with Na_2_SO_4_. The solvent was removed in vacuo and the residue was purified by flash chromatography on silica gel (at the gradient of 20% ethyl acetate in petroleum ether and ethyl acetate elution system) to give Bu-FOS 6.1 g as a colorless oil.

The synthesized Bu-FOS was analyzed by 400 MHz ^1^H NMR and 101 MHz ^13^C NMR, respectively.

^1^H NMR (400 MHz, CDCl_3_) δ 5.72 (t, *J* = 5.5 Hz, 2H), 5.54–5.40 (m, 3H), 5.36 (t, *J* = 6.4 Hz, 1H), 5.13 (t, *J* = 9.7 Hz, 1H), 4.97 (dd, *J* = 10.3, 3.7 Hz, 1H), 4.40–4.10 (m, 11H), 3.59 (q, *J* = 9.6 Hz, 2H), 2.43–2.13 (m, 22H, COCH_2_CH_2_CH_3_), 1.74–1.47 (m, 22H, COCH_2_CH_2_CH_3_), 1.02–0.81 (m, 33H, COCH_2_CH_2_CH_3_).

^13^C NMR (101 MHz, CDCl_3_) δ 173.22 (C=O), 173.13 (C=O), 172.98 (C=O), 172.56 (C=O), 172.53 (C=O), 172.43 (C=O), 172.38 (C=O), 172.33 (C=O), 172.25 (C=O), 172.06 (C=O), 171.95 (C=O), 103.17, 103.02, 89.17, 78.60, 77.70, 77.23, 76.24, 75.72, 74.46, 73.07, 69.71, 68.45, 67.82, 63.89, 63.00, 62.07, 61.95, 61.42, 35.95 (COCH_2_CH_2_CH_3_), 35.85 (COCH_2_CH_2_CH_3_), 35.79 (COCH_2_CH_2_CH_3_), 35.76 (COCH_2_CH_2_CH_3_), 35.72 (COCH_2_CH_2_CH_3_), 35.68 (COCH_2_CH_2_CH_3_), 35.64 (COCH_2_CH_2_CH_3_), 18.24 (COCH_2_CH_2_CH_3_), 18.22 (COCH_2_CH_2_CH_3_), 18.17 (COCH_2_CH_2_CH_3_), 18.12 (COCH_2_CH_2_CH_3_), 13.61 (COCH_2_CH_2_CH_3_), 13.58 (COCH_2_CH_2_CH_3_), 13.50 (COCH_2_CH_2_CH_3_).

### 2.4. Fermentations of FOS, Ac-FOS, and Bu-FOS In Vitro

Firstly, the basal medium was prepared according to the modified method described previously [19,20]. The basal medium (pH 7.4) contained (per liter) 4 g mice feeds (fat: 6.2%, carbohydrate: 35.6%, protein: 20.8%, and the calorific value: 17.6 KJ/g), 0.45 g of K_2_HPO_4_, 0.9 g NaCl, 0.45 g KH_2_PO_4_, 0.45 g (NH_4_)_2_SO_4_, 0.2 g MgSO_4_, 1.59 g NaHCO_3_, 0.02 g hemin, 0.5 g bile salts, 0.5 g L-cysteine hydrochloride, 2 mL Tween 80, 10 µL vitamin K and 4 mL 0.025% (*w*/*v*) resazurin solution and distilled water. The feces slurry was prepared according to the published method [21]. The fresh feces of five healthy mice (6–8 weeks, free of any treatment) were collected and stored at −80 °C before use. Equal amounts of feces from five mice were mixed and diluted with phosphate buffer (0.01 M, pH 7.4) to obtain the feces slurry (10%, *w*/*v*). Finally, 1 mL 10% feces slurry was added into 9 mL of a basal medium containing 0.1 M FOS, Ac-FOS, or Bu-FOS. After incubation at 37 °C for 12 h in an anaerobic condition, the samples were taken for further study. Each experiment was replicated four times independently.

### 2.5. pH and SCFAs Measurement

After fermentations for 12 h, the fermented broths were centrifuged (12,000× *g*, 4 °C for 10 min) and the supernatants were filtered through 0.22 µm membranes. The pH values were measured with a pH meter (Dolly scientific instrument Co., Ltd., Guangzhou, China). The concentrations of SCFAs were assayed in a similar method [22]. Briefly, 90 µL metaphosphoric acid (25%) and 810 µL supernatant were mixed. After 3 h of shaking, the mixtures were centrifuged at 4 °C (12,000× *g*, 10 min). Agilent 6890 N gas chromatography (GC) equipped with a DB-FFAP column (30 m × 250 µm × 0.25 µm) and an FID detector was used for SCFAs assay. The column temperature was heated at a rate of 20 °C/min from 60 °C to 220 °C and maintained for 1 min. Standard curves were constructed and acetate, propionate, butyrate levels were calculated.

### 2.6. Analysis of the Microbiota

The V3-V4 region of the gut bacteria was amplified using universal primer 338F/806R (338F: 5′-ACTCCTACGGGAGGCAGCAG-3′, 806R: 5′-GGACTACGVGGGTWTCTAAT-3′) by PCR for 16S rDNA analysis. The PCR products were then purified by a TIANGEN DNA gel purification kit (TIANgel Mini Purification Kit, TIANGEN, Beijing, China). MiSeq library construction and sequencing were performed using the Illumina MiSeq PE300 platform (Illumina, San Diego, CA, USA). The reads were filtered by QIIME (*Quantitative Insights into Microbial Ecology*, http://qiime.org/tutorials/processing_illumina_data.html (accessed on 10 February 2021)) quality filters. All the bioinformation data were analyzed on the free online platform of the Majorbio Cloud Platform (www.majorbio.com (accessed on 10 February 2021). All novel sequences were deposited at NCBI’s Sequence Read Archive under accession number SRP316090.

### 2.7. Antioxidant Activity Assay

#### 2.7.1. Scavenging Capacity of Hydroxyl Radical

A series of concentrations of FOS, Ac-FOS, and Bu-FOS dimethyl sulfoxide solutions (0.002, 0.004, 0.006, 0.008, and 0.01 mol/mL) were prepared to assay the scavenging capacity of hydroxyl radical according to the previous description [23]. Briefly, 150 µL 0.01 mol/L FeSO_4_ solution and 150 µL 0.01 mol/L salicylic acid-ethanol solution, and 150 µL sample solution were added to a tube containing 900 µL ddH_2_O. Then, 150 µL 10 mmol/L H_2_O_2_ solution was added to each tube and mixed evenly. After the reaction at 37 °C for 30 min, the absorbance at 510 nm was determined and recorded. The tubes containing 150 µL dimethyl sulfoxide instead of sample solution were used as the control group. In addition, the tubes with 150 µL water instead of H_2_O_2_ solution were used as the blank group. These experiments were performed three times for each group. The hydroxyl radical scavenging rate was calculated according to the following formula:

*Scavenging rate* (%) = [A − A_d_]/A_0_ × 100% (1)

A: absorbance of the sample solution; A_0_: the absorbance of the control solution; A_d_: the absorbance of the blank solution.

#### 2.7.2. Scavenging Capacity of Superoxide Anion

The scavenging capacity of hydroxyl radical was performed according to the previous method with appropriate modifications [24]. Briefly, a series of concentrations of FOS, Ac-FOS or Bu-FOS dimethyl sulfoxide solution (0.002, 0.004, 0.006, 0.008, and 0.01 mol/mL) were prepared. A volume of 2 mL 0.05 mol/L Tris-HCl buffer (pH 8.0) and 0.25 mL sample solution were mixed and incubated at 37 °C for 15 min. Then, 50 µL 10 mmol/L pyrogallol solution (preheated at 37 °C) was added to the mixture and shaken rapidly. Dimethyl sulfoxide instead of the sample solution was added as the control group. The absorbance at 420 nm was measured and recorded at 30 s and 240 s. These experiments were performed three times for each group. The scavenging capacity of hydroxyl radical was calculated by the following formula:

*Scavenging rate* (%) = [A_0_ − A]/A_0_ × 100% (2)

A_0_: absorbance of the control solution; A: absorbance of the sample solution.

### 2.8. Prebiotic Activity Analysis

According to the slightly modified method of Huang et al. [25], *L. rhamnosus* (CGMCC1.3724) and *B. longum* (CGMCC1.2186) were used for investigating the prebiotic activity of the Ac-FOS and Bu-FOS in vitro. Yeast Extract Peptone Dextrose Medium (YPD broth) (20 g/L glucose, 10 g/L yeast extract, 20 g/L peptone) with 0.01 mmol/mL FOS was prepared as the positive control. YPD broth containing FOS equivalents of glucose served as the blank control. In addition, the other two kinds of YPD broth with 0.01 mmol/mL Ac-FOS or Bu-FOS were also prepared. Overnight cultures of *L. rhamnosus* were inoculated into four modified YPD broths and incubated at 37 °C under anaerobic conditions. Bacterial counting was done by plate culture count and calculated as log (CFU/mL) after incubating for 24, 48, and 72 h. 

### 2.9. Statistical Analysis

All data are presented as the mean ± standard deviation. All graphs were plotted using Graphpad Prism 6.0 (GraphPad Software Inc., San Diego, CA, USA). Statistical analysis was performed with SPSS 20.0 (IBM, Armonk, NY, USA). One-way analysis of variance and Duncan’s multiple range tests were used to determine the differences between diverse samples, and *p* < 0.05 was considered statistically significant.

## 3. Results

### 3.1. Characterization of Esterified Fructo-Oligosaccharides

**Figure 1:**
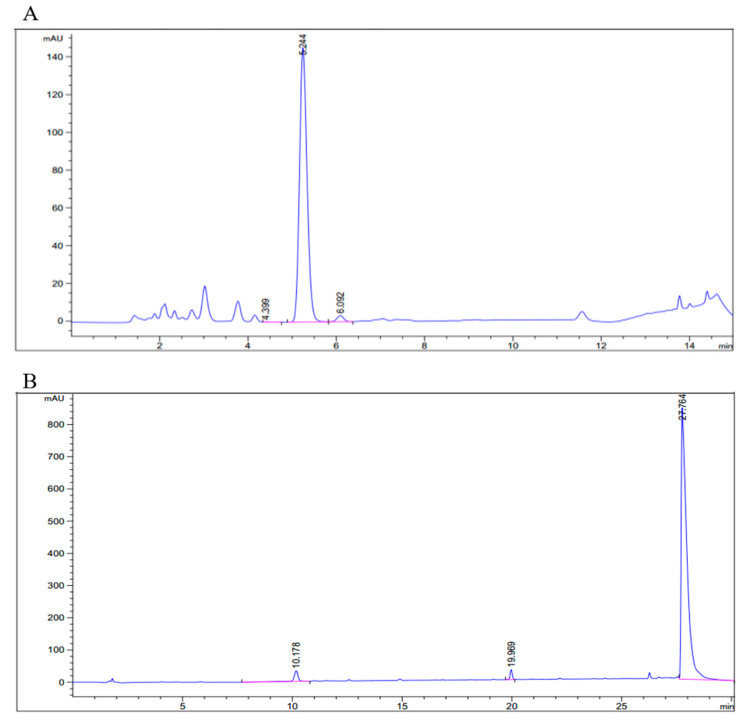
Purity test by HPLC-UV detector (Wavelength = 210 nm) for each esterified fructo-oligosaccharides. (**A**) Ac-FOS, (**B**) Bu-FOS.

High-performance liquid chromatography (HPLC) was used to measure the purity of synthesized esterified fructo-oligosaccharides. HPLC results indicated that esterified fructo-oligosaccharides showed excellent purity (>95%) (Figure 1). In addition, HPLC results guaranteed each synthesized compound was a single component.

**Figure 2:**
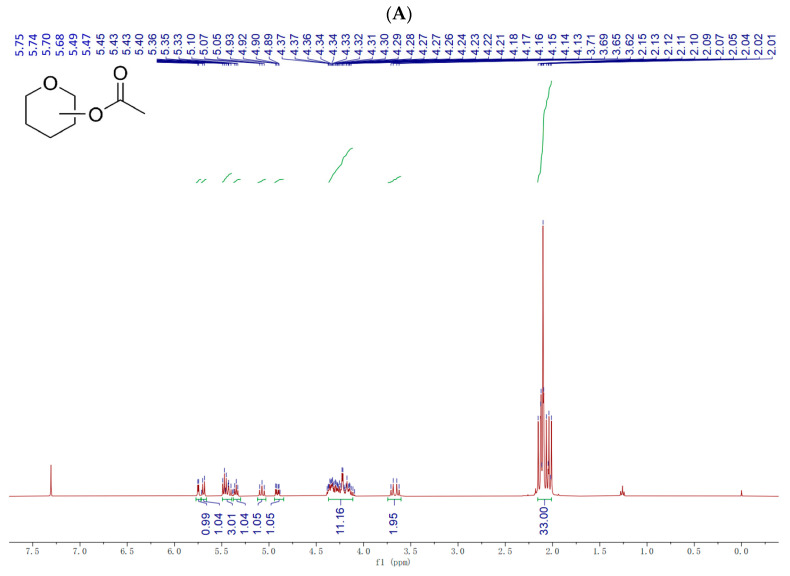

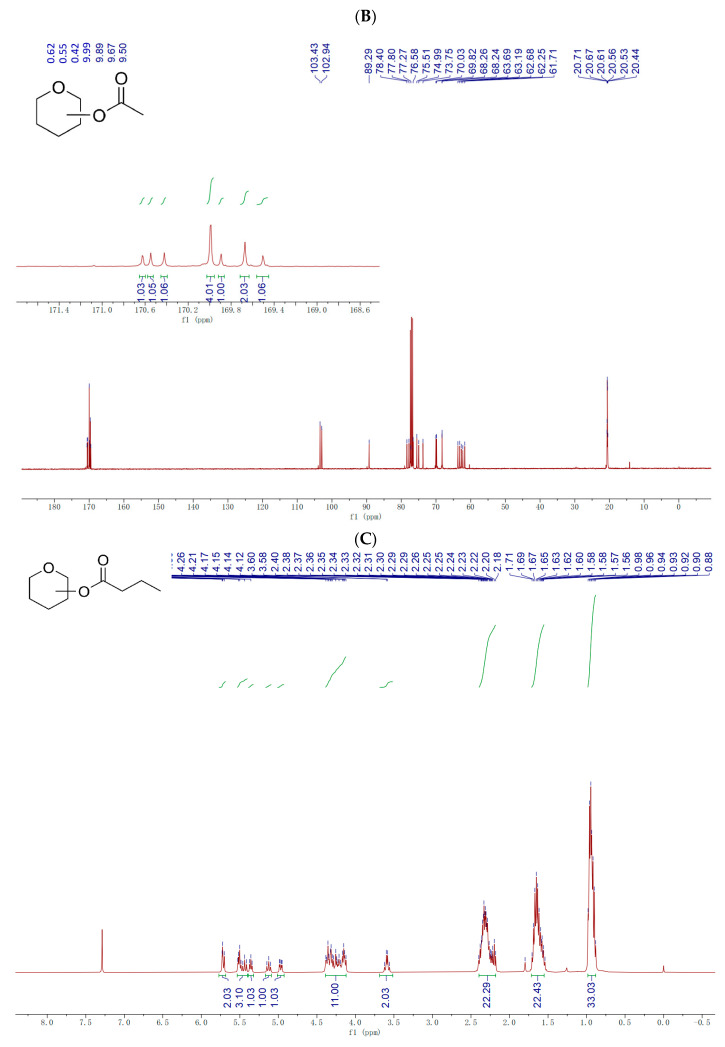

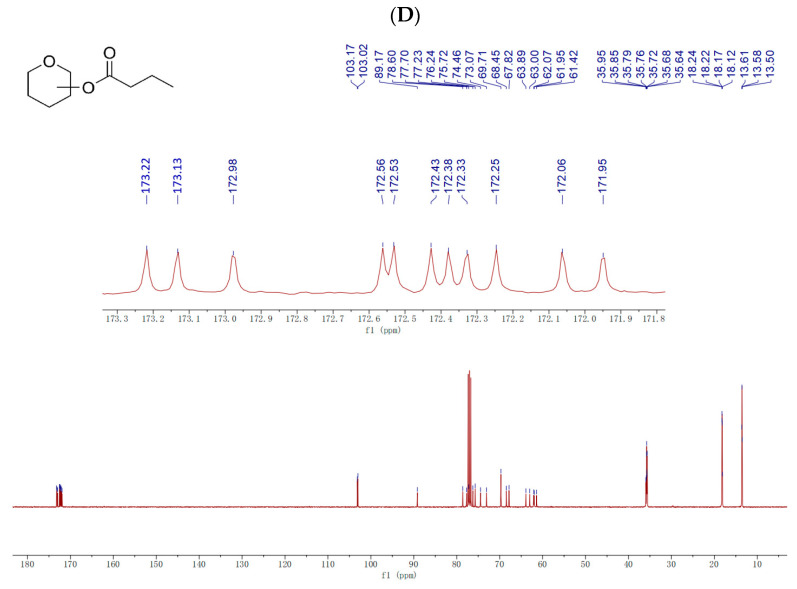
Nuclear magnetic resonance (NMR) spectra of esterified fructo-oligosaccharides. (**A**) ^1^H-NMR of Ac-FOS, (**B**) ^13^C-NMR of Ac-FOS, (**C**) ^1^H-NMR of Bu-FOS, and (**D**) ^13^C-NMR of Bu-FOS.

Nuclear magnetic resonance (NMR) experiments characterized the synthesized structure at the hydrogen and carbon level. As ^1^H-NMR of Ac-FOS is shown in Figure 2A, a chemical shift from 3.5 ppm to 6.0 ppm belongs to the carbohydrate region signal. 33H, which appeared at the chemical shift from 2.0 ppm to 2.8 ppm has the acetyl groups’ characteristic signal and led to the 11 (OC=OCH_3_) hydrogen signals. The result of the Ac-FOS ^13^C-NMR spectrum in Figure 2B is consistent with the result of its ^1^H-NMR spectrum. As shown in Figure 2B, 11 carbon signals ranging from 169 ppm to 171 ppm appeared in the spectrum, which reflect acetyl group (OC=OCH_3_) signals.

^1^H-NMR of Bu-FOS is shown in Figure 2C. A chemical shift from 3.5 ppm to 5.8 ppm indicates a carbohydrate region signal. 22H at the chemical shift from 2.1 ppm to 2.5 ppm, 22H at a chemical shift from 1.5 ppm to 1.8 ppm, and 33H at the chemical shift from 0.8 ppm to 1.0 ppm indicate butyryl group signals. The former consists of 11(OC=OCH_2_CH_2_CH_3_) hydrogen signals, the middle of them consists of 11 (OC=OCH_2_CH_2_CH_3_) hydrogen signals, and the latter consists of 14 (OC=O CH_2_CH_2_CH_3_) hydrogen signals. The result of the Bu-FOS ^13^C-NMR spectrum in Figure 2D is consistent with the result of its ^1^H-NMR spectrum. As shown in Figure 2D, 11 carbon signals ranging from 171 ppm to174 ppm appeared in the spectrum, which indicates propionyl group (OC=OCH_2_ CH_2_CH_3_) signals.

**Figure 3:**
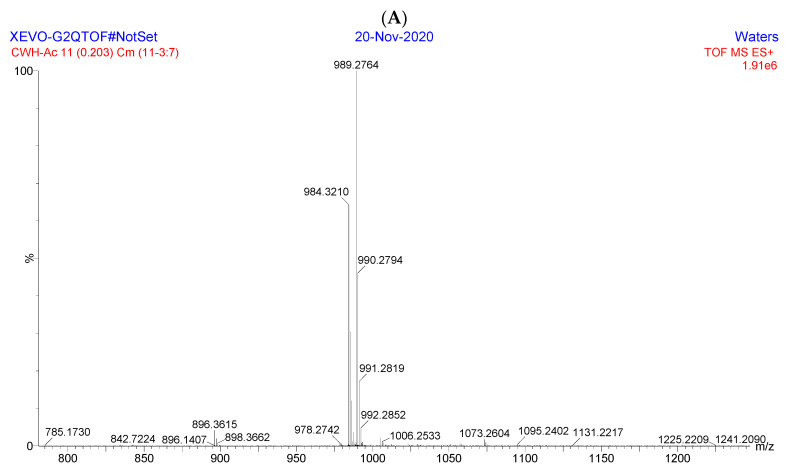

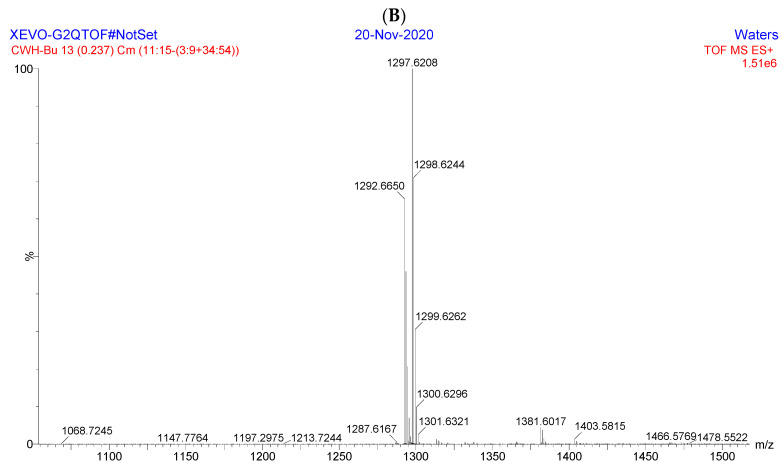
HRMS of esterified fructo-oligosaccharides. (**A**) HRMS of Ac-FOS, (**B**) HRMS of Bu-FOS.

High-resolution mass spectrometry (HRMS) experiments were used to acquire the exact mass and elemental composition of each esterified fructo-oligosaccharides. As shown in Figure 3A, HRMS (ESI) of Ac-FOS was calculated for C_40_H_58_NO_27_ [M + NH_4_]^+^as 984.3196 and found as 984.3210, while that calculated for C_40_H_54_O_27_Na [M + Na]^+^ was 989.2750 and found as 989.2764. As shown in Figure 3B, HRMS (ESI) of Bu-FOS calculated for C_62_H_102_NO_27_ [M + NH_4_]^+^ was 1292.6639, and found as 1292.6650, while that calculated for C_62_H_98_O_27_Na [M + Na]^+^ was1297.6193, and found as 1297.6208. The HRMS results indicated that 11 acetyl groups were found at Ac-FOS, and 11 butyryl groups were found at Bu-FOS. The results of HRMS were consistent with the NMR results.

### 3.2. pH Value and SCFAs Production

As shown in Table 1, the pH values of FOS, Ac-FOS, and Bu-FOS samples decreased from the initial pH of 7.20 to 4.66, 6.56, and 6.19, respectively. Compared to the FOS group, the Ac-FOS group increased the production of acetate by 26.35%. Although the concentration of acetate in FOS group and Bu-FOS group is not significant, the production of butyrate was 3.5025 mmol/mL in Bu-FOS group, while butyrate content was below our detection limit in FOS group. In addition, the concertation of the total SCFAs in Bu-FOS group was 4.22-fold in FOS group.

### 3.3. Microbiota Analysis

Extracted DNA samples from different groups in vitro were sequenced using MiSeq Illumina platforms (Illumina Inc., San Diego, CA, USA) generating a total of 526,210 sequence reads. The numbers of OTUs were 229 ± 11.71 for FOS group, 257 ± 52.66 for Ac-FOS group, and 229.3 ± 6.115 for Bu-FOS group. However, the difference between FOS and Ac-FOS, as well as, between FOS and Bu-FOS were not significant.

### 3.4. Analysis of Alpha Diversity Indices

The alpha diversity indices of the three groups are shown in Table 2. Chao1 indices reflected the species richness of the intestinal microbiota in the sample. The Shannon and Simpson indices reflected the diversity of the intestinal microbiota in the samples. The results showed all of the alpha diversity indices were not significant between FOS and Ac-FOS groups, as well as FOS and Bu-FOS groups.

### 3.5. Composition of Microbiota

#### 3.5.1. Differences in Phylum and Genus Level

**Figure 4:**
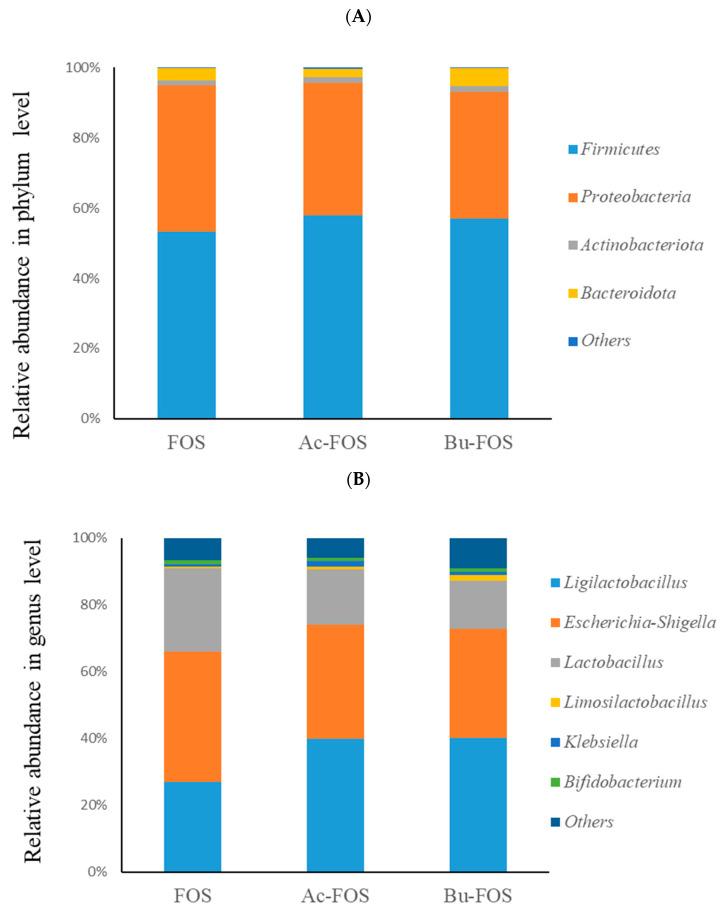
Relative abundance in phylum level and genus level. (**A**) Relative abundance in phylum level, (**B**) Relative abundance in genus level.

Figure 4A shows the relative abundance of dominant phylum and genus in FOS, Ac-FOS, and Bu-FOS groups. The FOS, Ac-FOS, and Bu-FOS samples had a similar dominant phylum, including Firmicutes, Bacteroidota, Proteobacteria, and Actinobacteriota. At the genus level, *Ligilactobacillus*, *Escherichia-Shigella*, *Lactobacillus*, *Limosilactobacillus*, *Klebsiella,* and *Bifidobacterium* were found in all samples (Figure 4B). Importantly, most of them have well-known benefits to host health.

#### 3.5.2. Differences in Species Level

**Figure 5:**
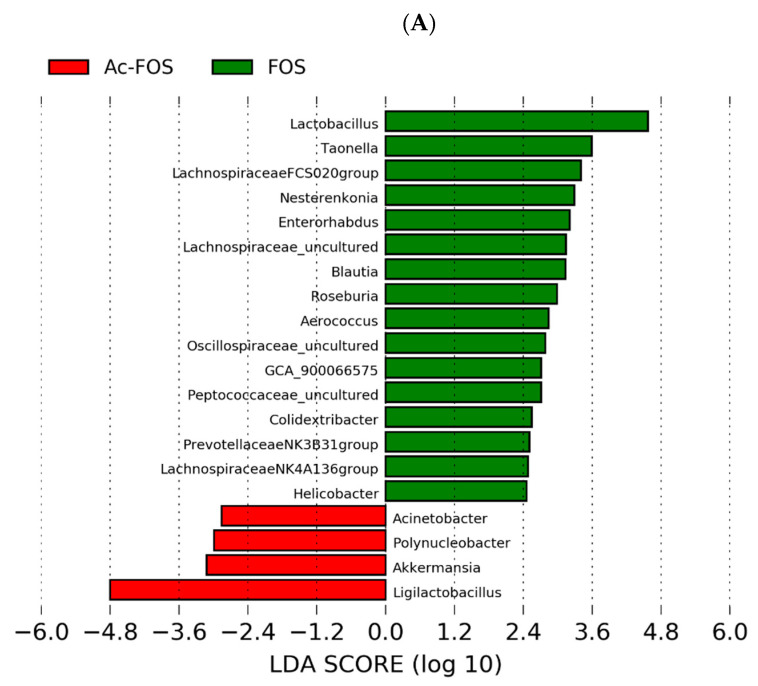

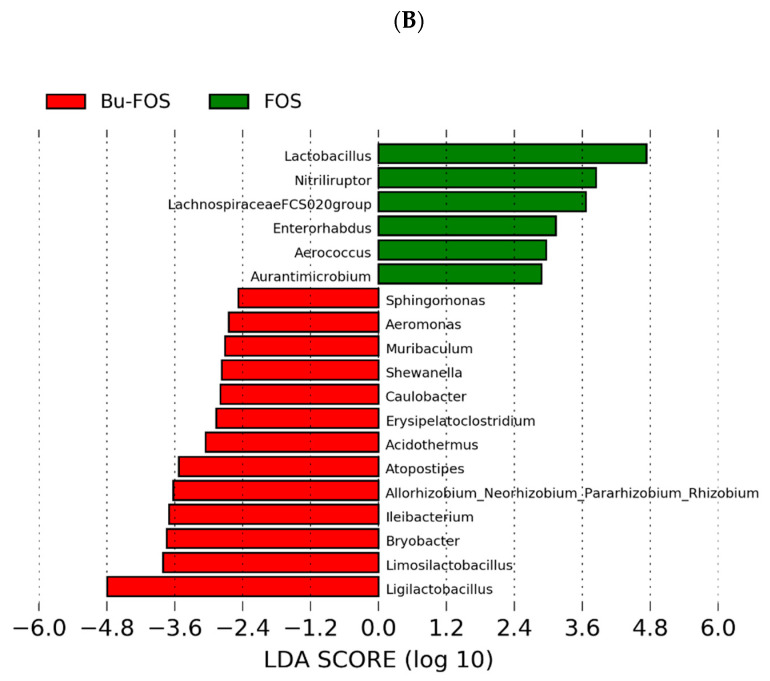
Comparison of relative abundance between different groups in genus level by LEfSe analysis. The items with LDA scores > 2.0 are listed, indicating a higher relative community abundance change in the corresponding group than in other groups. (**A**) FOS and Ac-FOS, (**B**) FOS and Bu-FOS.

The bacteria abundances of different groups seemed to be modulated by different treatments, and the differences were mainly displayed at the genus level. To identify the specific bacterial taxa in each group, the compositions of the microbiota from FOS, Ac-FOS, and Bu-FOS groups at the genus level were compared by LEfSe analysis (Score > 2.0). Compared with the Ac-FOS group, we found that 16 genera were significantly different in FOS group, while only four genera changed significantly in the Ac-FOS group (*Acinetobacter*, *Polynucleobacter*, *Akkermansia,* and *Ligilactobacillus*) (Figure 5A). As shown in Figure 5B, six genera changed significantly in the FOS group which included *Lactobacillus*, *Nitrilliruptor*, *Lachnospiraceae FCS020* group, *Enterrorhabdus*, *Aerococcus*, and *Aurantimicrobium*. However, 13 genera changed in FOS group mainly including *Ligilactobacillus*, *Limosilactobacillus*, *Bryobacter*, and *Ileibacterium*.

### 3.6. Analysis of Antioxidant Activity

#### 3.6.1. Analysis of Hydroxyl Radical Scavenging Ability

**Figure 6:**
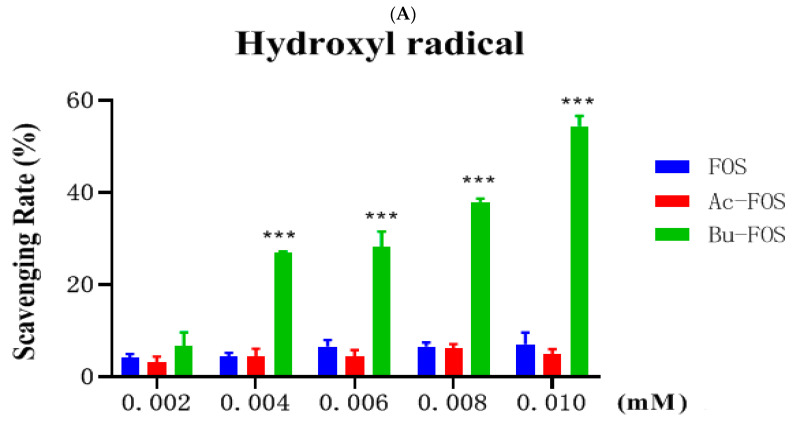

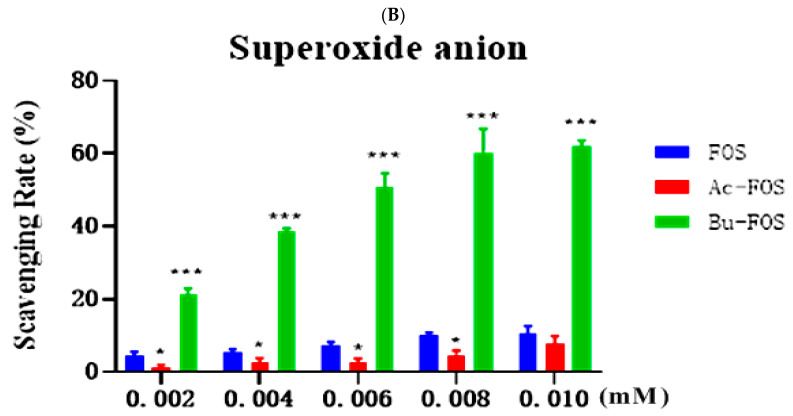
Antioxidant activity of FOS, Ac-FOS, and Bu-FOS. (**A**) Hydroxy radical activity, (**B**) Superoxide anion activity. “*”: *p* < 0.05, means changes in different concentrations.; “***”: *p* < 0.001, represents highly significant differences in different concentration gradients.

As shown in Figure 6A, the hydroxyl radical scavenging abilities of both FOS and Ac-FOS were lower than 10%, with no linear relationship in all concentrations. There was no significant difference between FOS and Ac-FOS in hydroxyl radical scavenging ability. However, a significant increase in hydroxyl radical scavenging rate could be observed in a dose-dependent manner with the Bu-FOS supplement, which was much higher than FOS and Ac-FOS at any concentration. At a concentration of 0.01 mol/mL, the hydroxyl radical scavenging ability reached 54.48%.

#### 3.6.2. Analysis of Superoxide Anion Scavenging Ability

The superoxide anion scavenging rate of FOS was lower than 10% at all gradient concentrations. The superoxide anion scavenging ability of Ac-FOS was slightly decreased with supplement increase. However, at all test concentrations, the superoxide anion scavenging rates of Ac-FOS were all lower than FOS. The superoxide anion scavenging rates of Bu-FOS were higher than 20% at all concentrations and significantly improved in a concertation-dependent manner. These could be increased to 61.98% when 0.01 mol/mL Bu-FOS was supplied (Figure 6B).

### 3.7. Prebiotic Activity

**Figure 7:**
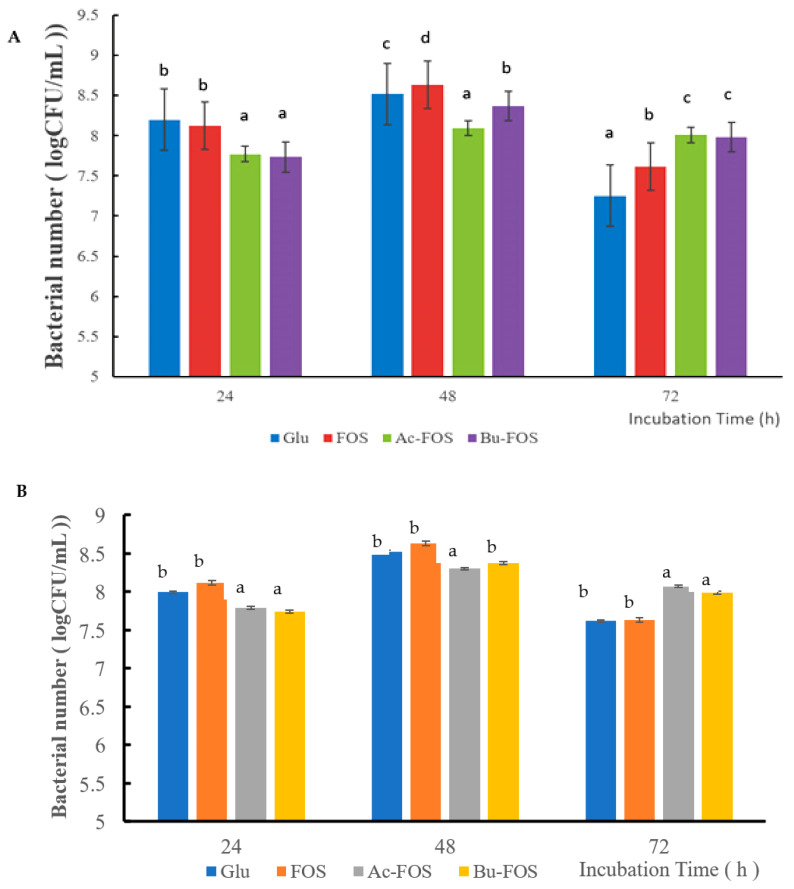
Prebiotic activity of FOS, Ac-FOS, and Bu-FOS on *L. rhamnosus* (**A**) and *B. longum* (**B**). Different lower-case letters in the same incubation time show significant differences between different treatments.

As shown in Figure 7A, when *L. rhamnosus* was incubated in different culture mediums with four supplements at 24 h, the number of live bacteria in the glucose group was higher than that of Ac-FOS and Bu-FOS groups. After 48 h incubation, the FOS group had the highest bacterial number of *L. rhamnosus*, while the bacteria numbers in Ac-FOS and Bu-FOS groups were still lower than that of glucose group. Although the numbers of viable bacteria in all four groups decreased after 72 h incubation, the Ac-FOS group and Bu-FOS had higher bacteria numbers than that FOS group and the glucose group. As shown in Figure 7B, the growth trend of *B. longum* was similar to that of *L. rhamnosus*.

## 4. Discussion

Non-digestible oligosaccharides are non-digestible by the host. Generally, they are bioavailable and beneficial for only a few species of bacteria such as probiotic bacteria, *Bifidobacteria*, and *Lactobacilli* to stimulate their growth. Previous reports stated the physiological functions of non-digestible oligosaccharides. Oligosaccharides decrease diarrhea by a decrease in gastrointestinal, respiratory, and urogenital tract infections. In addition, they can decrease cholesterol, triglyceride, and phospholipid concentrations in the blood to reduce the risks of diabetes, obesity, and colon cancer [26,27]. FOS play similar roles and have better prebiotic effects than polysaccharides. All the physiological functions are occur by adjusting pH, antioxidant activity, prebiotic activity, promoting SCFAs production, and microbiota changes. To further improve the prebiotic properties of FOS, acetylated and butyrylated fructo-oligosaccharides were synthesized and characterized based on NMR analysis. The prebiotic properties of Ac-FOS and Bu-FOS were investigated using mixed fresh feces fermentation experiments to obtain valuations.

After 12 h fermentation, the pH value of fermented broth was measured with a pH meter. We found that the pH values of Ac-FOS group and Bu-FOS group were increased from 4.66 ± 0.05 to 6.56 ± 0.08, and 6.16 ± 0.03, respectively, which changed within a reasonable range [28]. In addition, an increase of total SCFAs measured by gas chromatography was observed during a fermentation experiment in vitro. SCFAs concentrations and pH were inversely related and affected the growth of certain bacteria and types of metabolites produced during the fermentation [28,29]. As we know, butyrate, acetate, and propionate are the most important SCFAs that provide metabolic energy for the host and result in acidification of the bowel content [30,31]. Production of these acids is associated with the amelioration of some physiological health aspects, such as improvement of mineral absorption, lowering colon cancer risk, regulation of glucose and lipid metabolism and improving intestinal function [5]. Moreover, the concentration enhancements of acetate and butyrate were found in the Ac-FOS and Bu-FOS fermentation experiments separately; in particular, the concentration of total SCFAs was improved up to 4.22-fold. The increase of SCFAs concentration maybe because of SCFAs released from esterified-FOS caused by the hydrolysis of gut microbiota. A previous study reported that acetylated, propionylated or butyrylated starches increase large bowel short-chain fatty acids preferentially when fed to rats [32]. Clarke et al. study also showed that cooked butyrylated starch delivers esterified butyrate to the human colon effectively [33]. The greatest increase was in butyrate with corresponding increases of 460% in caecum [32]. Butyrate can resist inflammation reactions and strengthen epithelial barrier integrity. Butyrate in the intestine not only plays key functions in the colonic epithelium but also suppresses colon cancer [34,35,36]. Thus, supplement of butyrate may become a treatment strategy for gastrointestinal inflammation [37]. Butyrylated fructo-oligosaccharides provide a new approach for supplementing butyrate content in the intestine. Esterification of FOS may be a better system to deliver SCFAs, which maintain the benefits of FOS.

Antioxidant activity was evaluated by hydroxyl radical scavenging rate and superoxide anion scavenging rate measured by absorbance. Numerous modifications of polysaccharides including sulfation, phosphorylation, carboxymethylation, and selenization can boost antioxidant activities and may be effective in securing food, pharmaceutical, and cosmetic formulations. Feruloyl oligosaccharides strengthened the antioxidative capacity of the jejunum, as evidenced by increased contents of catalase, superoxide dismutase, glutathione peroxidase, and glutathione [38]. Although no differences in antioxidant activities were observed between FOS and Ac-FOS, excellent antioxidant activities were found when FOS was butyrylated. The hydroxyl radical scavenging rate of 0.01 mol/mL Bu-FOS supplement was increased by 55.39% while that of the FOS group was 3.44%. Superoxide anion scavenging rate of 0.01 mol/mL Bu-FOS supplement could be increased by 61.98% compared to 11.47% in the FOS group. The butyrylated treatment may be an effective method to enhance the antioxidant activity of fructo-oligosaccharides.

In the probiotic activity study, *L. rhamnosus* was used for investigating the prebiotic activity of the Ac-FOS and Bu-FOS in vitro. After 48 h incubation, FOS showed better activity to stimulate the growth of *L. rhamnosus* and *B. longum.* After 72 h incubation, the numbers of the living bacteria in Ac-FOS and Bu-FOS groups were greater than in the FOS group and Glu group. Phosphorylated polysaccharides had the strongest probiotic activity for *Lactobacillus bulgaricus* of all modified polysaccharides, and acetylated polysaccharides had significant growth-promoting effects for *Bifidobacterium adolescentis* [9]. Chemically modified prebiotics may be strain-selective for their growth-promoting activities. Compared with the control group and blank group, Ac-FOS and Bu-FOS showed a long period of stationary phase in bacterial growth, during which longer beneficial effects could be made. Long-term consumption could increase the effect of Ac-FOS and Bu-FOS.

Microbiota community differences were explored by 16s rDNA. Alpha diversity indices indicated no significant differences between FOS and Ac-FOS/Bu-FOS, which means that abundance and diversity of intestinal flora changed little after acetylated or butyrylated treatment of FOS. Firmicutes, Bacteroidota, Proteobacteria, and Actinobacteriota were the dominant phyla with no changes. Twenty genera significantly changed after the acetylated treatment, including *Acinetobacter*, *Polynucleobacter*, *Akkermansia*, *Ligilactobacillus,* among others. Nineteen genera changed significantly after the butyrylated treatment, including *Lactobacillus*, *Nitrilliruptor*, *Lachnospiraceae FCS020* group, *Enterrorhabdus*, *Aerococcus*, and *Aurantimicrobium*. *Ligilactobacillus*, *Limosilactobacillus*, *Bryobacter*, and *Ileibacterium*. Overall, as we expected, beneficial bacteria such as *Ligilactobacillus*, *Escherichia-Shigella*, *Lactobacillus*, *Limosilactobacillus*, *Klebsiella,* and *Bifidobacterium* were found in all groups at the genus level. Similarly, acetylated and butyrylated fructo-oligosaccharides also increased the beneficial bacteria and played a better role in intestinal microbiota regulation.

## 5. Conclusions

In this work, Ac-FOS and Bu-FOS were successfully synthesized and characterized as candidate prebiotics. The bioactivities of two modified FOS were changed and the content of SCFAs were effectively increased. Especially, the antioxidant activity of Bu-FOS was highly improved. Thus, esterification of FOS, especially Bu-FOS, can lead to potential prebiotic supplements that may be developed into dietary supplements for SCFA delivery.

## Figures and Tables

**Table 1:** Changes in SCFAs production and pH by 12 h anaerobic fermentation of feces samples (mmol/mL).

Sample	Acetate	Propionate	Butyrate	Total SCFAs	pH
FOS Group	0.8293 ± 0.1147 ^b^	ND	ND	0.8293 ± 0.1147 ^c^	4.66 ± 0.05 ^c^
Ac-FOS Group	1.0478 ± 0.1340 ^a^	ND	ND	1.0478 ± 0.1340 ^b^	6.56 ± 0.08 ^a^
Bu-FOS Group	0.8142 ± 0.0360 ^b^	ND	2.6882 ± 0.1187	3.5025 ± 0.1286 ^a^	6.16 ± 0.03 ^b^

The data shown are mean ± SD from four independent experiments. Different lower-case superscript letters in the same column show significant differences between different groups, respectively. “ND”: not detected.

**Table 2:** Analysis of alpha diversity indices.

	FOS Group	Ac-FOS Group	Bu-FOS Group
Shannon	2.313 ± 0.203	2.285 ± 0.305	2.453 ± 0.183
Simpson	0.1689 ± 0.0197	0.1787 ± 0.0524	0.1591 ± 0.018
Chao 1	264.3 ± 28.7	280.5 ± 145.5	260.8 ± 16.2

The data shown are mean ± SD from three independent experiments.

## Data Availability

The data presented in this study are available in the article.
